# Association between the non-HDL-cholesterol to HDL-cholesterol ratio and non-alcoholic fatty liver disease in Chinese children and adolescents: a large single-center cross-sectional study

**DOI:** 10.1186/s12944-020-01421-5

**Published:** 2020-11-22

**Authors:** Shouxing Yang, Jinwei Zhong, Mengsi Ye, Lei Miao, Guangrong Lu, Changlong Xu, Zhanxiong Xue, Xinhe Zhou

**Affiliations:** 1 Department of Gastroenterology, The Second Affiliated Hospital and Yuying Children’s Hospital of Wenzhou Medical University, Wenzhou, 325000 Zhejiang China; 2 Department of Endocrinology, The Second Affiliated Hospital and Yuying Children’s Hospital of Wenzhou Medical University, Wenzhou, 325000 Zhejiang China

**Keywords:** Non-alcoholic fatty liver disease, NHDLC/HDLC ratio, Children, Adolescents, Cross-sectional study, Receiver operating characteristic curve

## Abstract

**Background:**

The non-HDL-cholesterol to HDL-cholesterol (NHDLC/HDLC) ratio is closely related to a variety of dyslipidemia-related diseases. This study examined the relationship between the NHDLC/HDLC ratio and non-alcoholic fatty liver (NAFLD) in children and adolescents.

**Methods:**

This cross-sectional survey included a total of 7759 eligible Chinese children and adolescents (5692 boys and 2067 girls) who received routine medical examinations. The anthropometric and laboratory data of the subjects were collected. NAFLD was diagnosed by liver ultrasonography. Binary logistic regression analysis was performed on the NHDLC/HDLC ratio, NHDLC, HDLC and NAFLD. Receiver operating characteristic (ROC) curve analysis was used to compare the diagnostic significance of the above parameters for NAFLD.

**Results:**

The total prevalence of NAFLD was 4.36%, and the prevalence in boys was higher than that in girls (5.61% vs. 1.9%, *P* < 0.001). The prevalence of NAFLD was positively correlated with the NHDLC/HDLC ratio (*P* < 0.001). The binary logistic regression analysis demonstrated that the OR was 8.61 (95% CI, 5.90–12.57, *P* < 0.001) in tertile 3 (highest NHDLC/HDLC ratio) compared with tertile 1 (lowest NHDLC/HDLC ratio). After adjustment for age, sex, body mass index (BMI), alanine aminotransferase (ALT), uric acid (UA), total bilirubin (TB), fasting plasma glucose (FPG) and Homeostasis Model Assessment of Insulin Resistance (HOMA-IR), the OR for tertile 3 (OR = 1.83, 95% CI, 1.04–3.22, *P* = 0.035) was still significantly higher than that of tertile 1. The area under the curve (AUC) of the NHDLC/HDLC ratio of boys was 0.787, which was significantly greater than NHDLC and HDLC (0.719 and 0.726, *P* < 0.001). For girls, the AUC of the NHDLC/HDLC ratio was 0.763, which was also significantly greater than NHDLC (0.661, *P* < 0.001). The cutoff point of the NHDLC/HDLC ratio was 2.475 in boys and 2.695 in girls. In addition, the AUC of the NHDLC/HDLC ratio was 0.761 in subjects with normal ALT levels (ALT ≤40 U/L), which was significantly higher than NHDLC (0.680, *P* < 0.001) and HDLC (0.724, *P* = 0.007). For subjects with elevated ALT levels (ALT > 40 U/L), the AUC of the NHDLC/HDLC ratio (0.746) was also significantly greater than NHDLC (0.646, *P* < 0.001).

**Conclusions:**

The NHDLC/HDLC ratio was positively correlated with NAFLD in Chinese children and adolescents. It may serve as an effective indicator to help identify NAFLD in children and adolescents.

## Background

Non-alcoholic fatty liver disease (NAFLD) is a chronic liver disease (CLD) involving fatty liver, liver steatosis and nonalcoholic steatohepatitis (NASH) [1]. Currently, NAFLD is the major reason for CLD in the pediatric population. Given the epidemiological data, the prevalence of NAFLD is 2.6–7.1% of all children and approximately 27.8–41.2% of obese children. Furthermore, the incidence ratio of NAFLD is on the rise [2, 3].

NAFLD is recognized as metabolic syndrome (MS) that manifested in the hepatic area, and its progressive form NASH increases the risk of liver cancer, end-stage liver disease and cirrhosis [4]. Pediatric NAFLD is considered to be involved in the pathogenesis of diabetes mellitus (DM), MS and cardiovascular disease (CVD) [5]. Since children with NAFLD are usually asymptomatic, the diagnosis of NAFLD in childhood and adolescence is challenging. Although the diagnostic gold standard for NAFLD is liver biopsy, it is not readily accepted by children because of its invasive nature. Liver ultrasonography is a feasible and noninvasive means of diagnosing NAFLD, but not all children undergo liver ultrasonography because their parents may be reluctant to let them receive medical examination for a clinically silent disease [6]. Therefore, valuable indicators for NAFLD should be identified for early detection and prevention of later progression.

The non-HDL-cholesterol to HDL-cholesterol (NHDLC/HDLC) ratio is closely related to a variety of dyslipidemia-related diseases, such as chronic kidney disease, gallbladder polyp, insulin resistance (IR) and MS. In addition, the NHDLC/HDLC ratio has a better predictive value for CVD in type 2 DM than high-density lipoprotein cholesterol (HDLC) [7–10]. A recent prospective study found that the NHDLC/HDLC ratio is more sensitive for predicting NAFLD in adults than non-high-density lipoprotein cholesterol (NHDLC) [11]. However, the association between the NHDLC/HDLC ratio and NAFLD in childhood and adolescence remains unclear. Therefore, This study examined the relationship between the NHDLC/HDLC ratio and NAFLD in childhood and adolescence.

## Materials and methods

### Subjects

A total of 7759 children and adolescents who received regular medical examinations from January 2015 to January 2020 in the Second Affiliated Hospital of Wenzhou Medical University were investigated. The inclusion criteria were subjects aged 2–18 years who had liver ultrasonography. Patients with hepatitis virus infection, metabolic liver disease, lipid-lowering agent treatment in the last month, hereditary hyperlipidemia or regular alcohol consumption were excluded.

### The elements of anthropometry and laboratory examinations

The clinical and anthropometric measurements assessed were age, sex, weight and height. Height and weight were examined on the same day. Body weight (kg) divided by height squared (m^2^) was used to calculate the body mass index (BMI). This study used fasting blood samples to assess metabolic variables using standard laboratory testing. Data collected for this study included total cholesterol (TC), fasting plasma glucose (FPG), HDLC, triglycerides (TG), alanine aminotransferase (ALT), alkaline phosphatase (APL), glycated hemoglobin A1c (HbA1c), low-density lipoprotein cholesterol (LDLC), albumin (ALB), uric acid (UA), aspartate aminotransferase (AST), gamma-glutamyl transferase (GGT), total bilirubin (TB) and creatinine (Cr). Homeostasis Model Assessment of IR (HOMA-IR) was calculated by HOMA-IR = FPG × fasting insulin/22.5 [12]. Children with BMI > 95th percentile were classified as obese [13].

### Ultrasonography

Liver ultrasonography was performed by an experienced expert with a Philips En2visor26 type ultrasonic diagnostic instrument (probe frequency 3.5–5.0 M*Hz*). The diagnosis of NAFLD was made according to the following ultrasonic criteria: ultrasound signals suggesting deep attenuation, vascular blurring, and discursively expanded echo (bright) liver showing echoes on the kidney or spleen [14].

### Statistical analysis

All data were evaluated using SPSS 21.0 (SPSS Inc., Chicago, IL, USA) and EmpowerStats (http://www.empowerstats.com). Means ± standard deviations (SD) denoted continuous data. Categorical variables were described using percentages. All participants were stratified into three tertiles according to the NHDLC/HDLC ratio (≤1.79, 1.80–2.36, ≥2.37). To evaluate the differences between groups, this study performed one-way ANOVA followed by the least significant difference (LSD) test on continuous variables with Gaussian distribution. The chi squared (χ^2^) test was performed on the categorical data. Binary logistic regression analysis was performed to evaluate the association between the NHDLC/HDLC ratio and NAFLD. The NHDLC/HDLC ratio was calculated as medians with interquartile ranges (IQR; IQR = Q3–Q1, where Q3 is the third quartile and Q1 is the first quartile of data distribution). The potential confounders used for adjustment included age, sex, BMI, ALT, UA, TB, FPG and HOMA-IR. Receiver operating characteristic (ROC) curve analysis was performed to evaluate the effectiveness of different lipid levels as indicators for NAFLD. The *P* value for a two-tailed test < 0.05 reflected statistical significance, and the figures were generated by GraphPad Prism 6 (GraphPad Software Inc., San Diego, CA, USA).

## Results

### Baseline characteristics

**Fig. 1:**
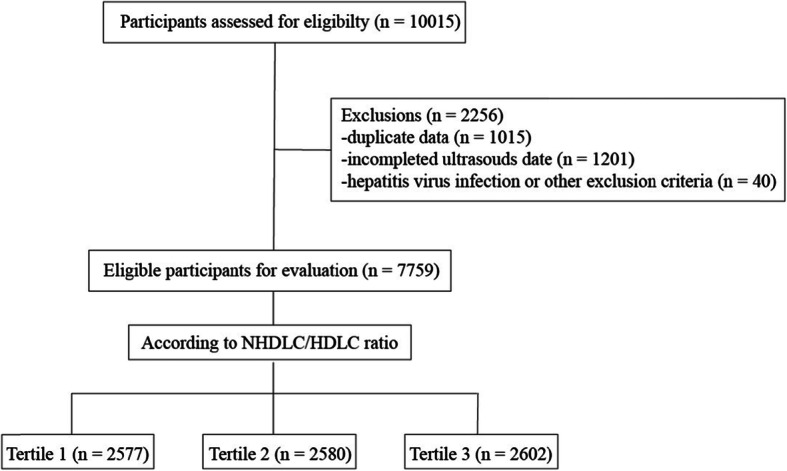
Flowchart of participant selection for the study

As shown in Fig. 1, a total of 7759 eligible children and adolescents (5692 boys, 2067 girls) were recruited. The mean age of the participants was 9.09 ± 3.30 years, and the mean BMI was 17.38 ± 3.72 kg/m^2^. Table 1 compares the baseline characteristics by tertiles of the NHDLC/HDLC ratio. These three tertiles were significantly different. In contrast to subjects in tertile 3 (highest NHDLC/HDLC ratio), subjects in tertile 1 (lowest NHDLC/HDLC ratio) had lower BMI, ALT, GGT, UA, ALB, TC, TG, LDLC, HbA1c (%), FPG and HOMA-IR and higher TB, ALP and HDLC (*P* < 0.001). The incidence of obesity noticeably increased across NHDLC/HDLC tertiles 1, 2 and 3 (4.81, 9.22 and 21.18%, respectively, *P* < 0.001).

**Table 1:** Baseline characteristics of participants

Characteristics	NHDLC/HDLC ratio			*P* value
	Tertile 1	Tertile 2	Tertile 3	
	(≤ 1.79)	(1.80–2.36)	(≥ 2.37)	
Sample size	2577	2580	2602	
Age (year)	9.24 ± 3.11	9.00 ± 3.31^b^	9.03 ± 3.47	0.017
Sex, n (%)				< 0.001
Girl	771 (29.92)	950 (36.82)^b^	906 (34.82)^a^	
Boy	1806 (70.08)	1630 (63.18)^b^	1696 (65.18)^a^	
BMI (kg/m^2^)	16.47 ± 2.64	17.02 ± 3.27^b^	18.64 ± 4.60^a/c^	< 0.001
ALT (U/L)	15.12 ± 8.49	15.93 ± 17.55	19.27 ± 19.88^a/c^	< 0.001
AST (U/L)	26.51 ± 6.75	26.64 ± 13.29	27.09 ± 13.62	0.175
ALP (U/L)	252.60 ± 78.46	242.84 ± 71.71^b^	240.50 ± 76.05^a^	< 0.001
GGT (U/L)	12.29 ± 5.27	12.83 ± 6.02^b^	15.45 ± 9.98^a/c^	< 0.001
TB (mmol/L)	9.88 ± 4.38	9.66 ± 4.36	9.27 ± 4.14^ac^	< 0.001
Cr (μmol/L)	39.50 ± 8.75	39.31 ± 9.48	39.35 ± 10.24	0.749
UA (μmol/L)	300.55 ± 69.92	305.06 ± 73.84	327.42 ± 85.44^a/c^	< 0.001
ALB (g/L)	45.36 ± 2.22	45.48 ± 2.17	45.78 ± 2.33^a/c^	< 0.001
TC (mmol/L)	4.09 ± 0.63	4.27 ± 0.65^b^	4.62 ± 0.82^a/c^	< 0.001
TG (mmol/L)	0.77 ± 0.30	0.89 ± 0.36^b^	1.21 ± 0.64^a/c^	< 0.001
HDLC (mmol/L)	1.67 ± 0.29	1.39 ± 0.22^b^	1.17 ± 0.21^a/c^	< 0.001
LDLC (mmol/L)	1.90 ± 0.45	2.31 ± 0.46^b^	2.79 ± 0.67^a/c^	< 0.001
HbA1c (%)	5.43 ± 0.29	5.43 ± 0.27	5.46 ± 0.29^a/c^	0.001
FPG (mmol/L)	4.68 ± 0.39	4.70 ± 0.38	4.73 ± 0.37^a/c^	< 0.001
HOMA-IR	1.42 ± 1.28	1.67 ± 1.93^b^	2.16 ± 2.07^a/c^	< 0.001
Obesity [n (%)]	124 (4.81%)	238 (9.22%)^b^	551 (21.18%)^a/c^	< 0.001

Data are expressed as the mean ± SD or percentage

*BMI* Body mass index, *ALT* Alanine aminotransferase, *AST* Aminotransferase, *ALP* Alkaline phosphatase, *GGT* Gamma-glutamyl transferase, *TB* Total bilirubin, *Cr* Creatinine, *UA* Uric acid, *ALB* Albumin, *TC* Total cholesterol, *TG* Triglycerides, *HDLC* High-density lipoprotein cholesterol, *LDLC* Low-density lipoprotein cholesterol, *HbA1c* Glycated hemoglobin A1c, *FPG* Fasting plasma glucose, *HOMA-IR* Homeostasis Model Assessment of Insulin Resistance

^a^*P* < 0.05, tertile 3 compared with tertile 1

^b^*P* < 0.05, tertile 2 compared with tertile 1

^c^*P* < 0.05, tertile 3 compared with tertile 2

### Prevalence of NAFLD and the NHDLC/HDLC ratio

**Fig. 2:**
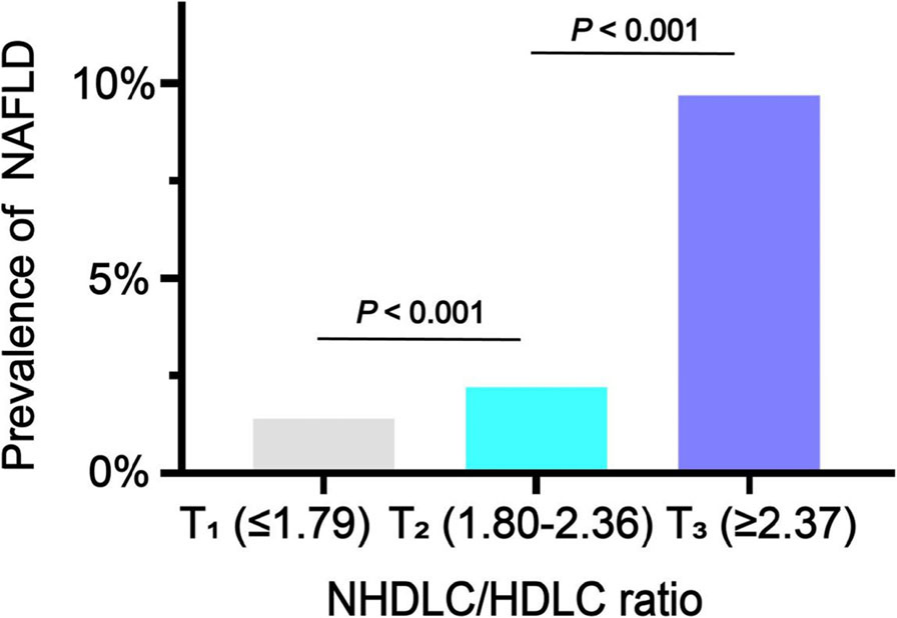
The prevalence of NAFLD according to the NHDLC/HDLC ratio tertiles

**Fig. 3:**
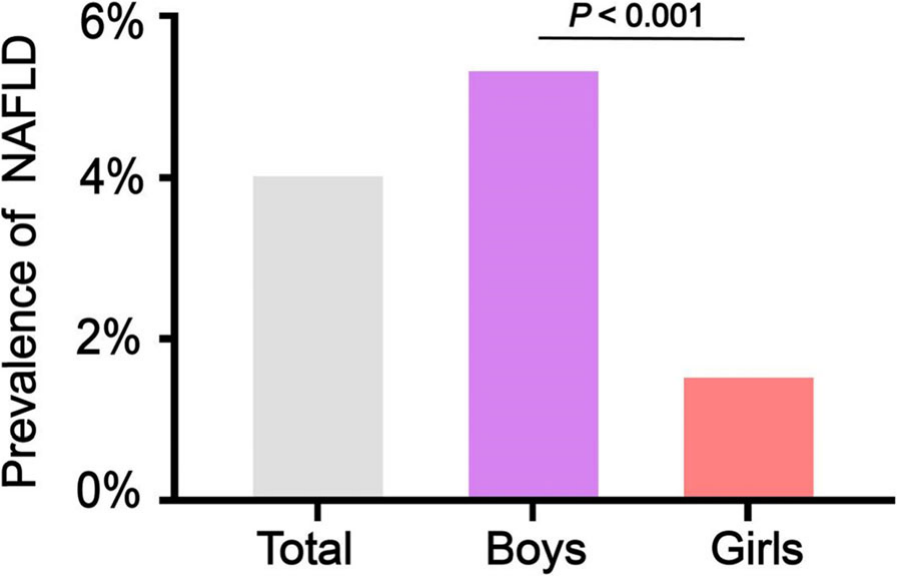
The prevalence of NAFLD in boys compared to girls

The prevalence of NAFLD was positively correlated with the NHDLC/HDLC ratio. Tertile 2 (2.33%, *P* < 0.001) and tertile 3 (9.49%, *P* < 0.001) showed a significantly higher NAFLD prevalence compared with tertile 1 (1.2%). The prevalence of NAFLD in tertile 3 was nearly eight times higher than tertile 1 (Fig. 2). In addition, the total prevalence of NAFLD was 4.36%, and the prevalence for boys was higher than girls (5.61% vs. 1.9%, *P* < 0.001) (Fig. 3).

### Association between the NHDLC/HDLC ratio and NAFLD

The binary logistic regression analysis showed that the odds ratio (OR) of NAFLD increased significantly with the increase in the NHDLC/HDLC ratio. The OR was 8.61 (95% CI, 5.90–12.57, *P* < 0.001) in tertile 3 compared with tertile 1. When potential confounders (age, sex, BMI, ALT, UA, TB, FPG and HOMA-IR) were adjusted, the OR for tertile 3 (OR = 1.83, 95% CI, 1.04–3.22, *P* = 0.035) was still significantly higher than tertile 1. Moreover, with the per IQR increase in the NHDLC/HDLC ratio, the OR for NAFLD was 1.89 (95% CI, 1.52–2.34, *P* < 0.001). (Table 2).

**Table 2:** ORs for NAFLD by NHDLC/HDLC ratio

NHDLC/HDLC ratio	Crude			Adjusted^a^		
	β	OR (95% CI)	*P*	β	OR (95% CI)	*P*
Tertile 1 (≤1.79)		1			1	
Tertile 2 (1.80–2.36)	0.67	1.96 (1.16–3.03)	0.002	0.07	1.07 (0.57–2.04)	0.824
Tertile 3 (≥2.37)	2.15	8.61 (5.90–12.57)	0.000	0.61	1.83 (1.04–3.22)	0.035
Per IQR (=0.9)	1.06	2.88 (2.58–3.22)	0.000	0.63	1.89 (1.52–2.34)	0.000

Data are coefficient (β), odds ratio (OR) and 95% confidence intervals (CI), *P* value

*NAFLD* Non-alcoholic fatty liver disease, *NHDLC/HDLC* Non-HDL-cholesterol to HDL-cholesterol, *IQR* Interquartile range

Adjusted^a^ for age, sex, BMI, ALT, UA, TB, FPG and HOMA-IR

### The diagnostic significance of the NHDLC/HDLC ratio for NAFLD

**Fig. 4:**
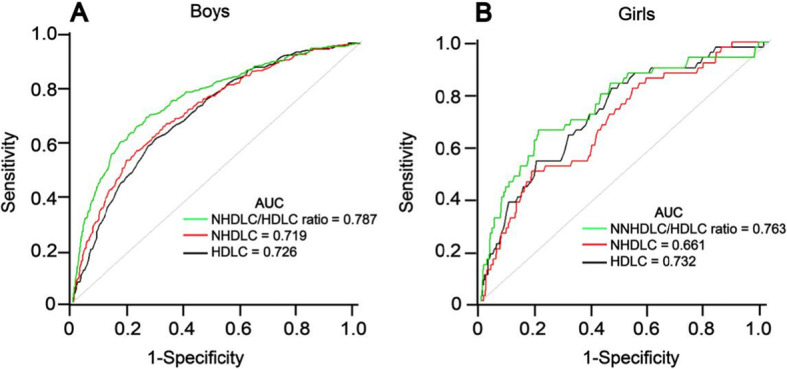
ROC curves of NHDLC/HDLC ratio, HDLC and NHDLC for NAFLD in boys (**a**) and girls (**b**)

**Fig. 5:**
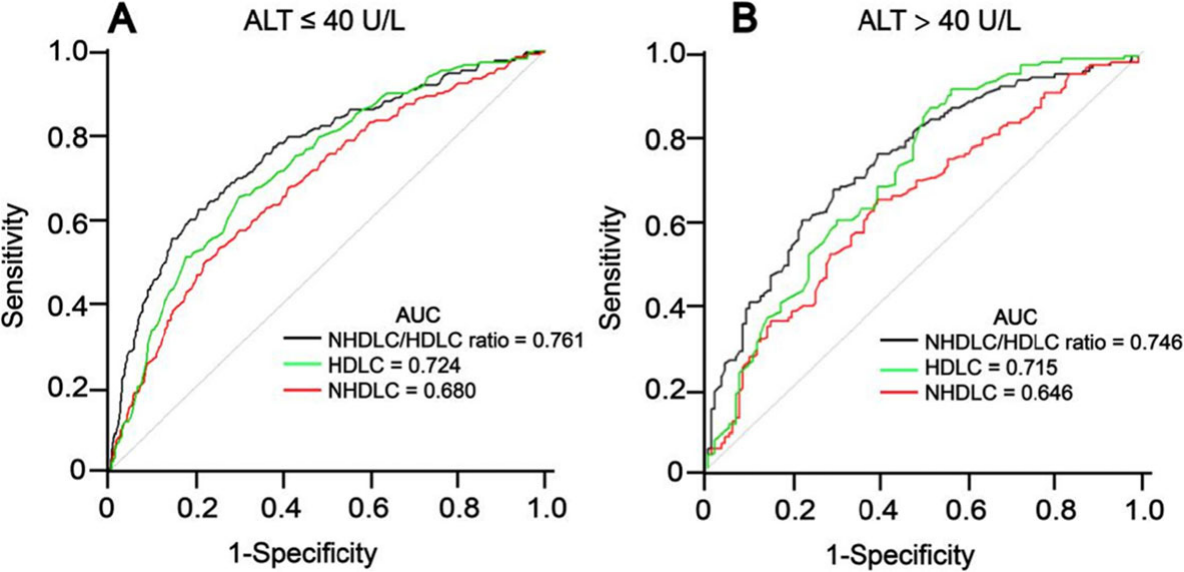
ROC curves of NHDLC/HDLC ratio, HDLC and NHDLC for NAFLD in subjects with normal ALT levels (**a**) and elevated ALT levels (**b**)

To assess the diagnostic significance of the NHDLC/HDLC ratio, NHDLC and HDLC for NAFLD, this study used sex-specific ROC curve analysis (Fig. 4). The area under the curve (AUC) of the NHDLC/HDLC ratio was 0.787 (0.758–0.816) in boys, which was significantly greater than NHDLC (0.719, 0.687–0.751, *P* < 0.001) and HDLC (0.726, 0.698–0.755, *P* < 0.001). For girls, the AUC of the NHDLC/HDLC ratio (0.763, 0.688–0.837) was also significantly greater than NHDLC (0.661, 0.580–0.743, *P* < 0.001), but there was no considerable difference from HDLC (0.732, 0.663–0.802, *P* = 0.239). The cutoff point of the NHDLC/HDLC ratio in boys was 2.475, exhibiting a sensitivity of 71.18% and a specificity of 74.46%. The cutoff point in girls was 2.695, exhibiting a sensitivity of 66% and a specificity of 80.09%. Besides, Fig. 5 shows the ROC curves for the different levels of ALT. The AUC of the NHDLC/HDLC ratio was 0.761 (0.725–0.798) in subjects with normal ALT levels (ALT ≤40 U/L), which was significantly greater than NHDLC (0.680, 0.642–0.718, *P* < 0.001) and HDLC (0.724, 0.689–0.758, *P* = 0.007). For subjects with elevated ALT levels (ALT > 40 U/L), the AUC of the NHDLC/HDLC ratio (0.746, 0.687–0.806) was also significantly greater than NHDLC (0.646, 0.579–0.713, *P* < 0.001), but there was no considerable difference with HDLC (0.715, 0.652–0.778, *P* = 0.226).

## Discussion

This study is the first to find that a high NHDLC/HDLC ratio was highly positively correlated with the prevalence of NAFLD in childhood and adolescence. Moreover, the diagnostic value of the NHDLC/HDLC ratio for NAFLD was better than NHDLC in both boys and girls. Therefore, the NHDLC/HDLC ratio may assist in the early identification of NAFLD in childhood and adolescence.

Previous studies confirmed that MS and obesity were the major risk elements for NAFLD in the pediatric population and dyslipidemia played a pivotal role in NAFLD pathogenesis [4, 15]. Recent clinical assessments suggested that statin-based therapies (lipid-lowering agent treatment) improved impaired hepatic function in patients with NAFLD [16]. The dyslipidemia in NAFLD is symbolized by increased levels of TG and decreased levels of HDLC [17]. Driven by lipid metabolism abnormalities, excess fat in NAFLD accumulates in hepatocytes. The mentioned intrahepatic lipid accumulation was attributed to decreased TG export, uptake of liver free fatty acids (FFAs) and very low-density lipoprotein cholesterol production [18, 19]. Ectopic lipid overloading in hepatocytes is associated with inflammation induction, oxidative stress, and the secretion of numerous cytokines (including adiponectin, interleukins and tumor necrosis factor) [20]. Moreover, excess FFAs form fatty acyl-CoAs sequentially catalyzed by fatty acyl-CoA synthetase, which likely induces β-oxidation pathways. The above inflammation and oxidative stress are involved in NAFLD initiation and progression [21, 22].

NHDLC, which refers to TC minus HDLC, is regarded as a secondary target of the lipid-lowering therapeutic method [23]. Compared to NHDLC, the NHDLC/HDLC ratio covers more comprehensive abilities of lipid dysregulation and a better capacity to assess lipid metabolism-related disease risk. According to the existing study, the NHDLC/HDLC ratio was better than NHDLC in predicting the occurrence of CVD in type 2 DM [10], and the ratio exhibited a higher prediction result than the apoB/apoA1 ratio of IR and MS [9]. A prospective cohort study revealed that the NHDLC/HDLC ratio outperformed NHDLC in predicting new-onset NAFLD in the Chinese adult population [11]. Therefore, it is necessary to examine the association between the NHDLC/HDLC ratio and NAFLD in the pediatric population.

Baseline characteristic data of 7759 subjects were stratified into three tertiles according to the NHDLC/HDLC ratio, and the results showed that laboratory variables (such as ALT, TG, LDLC, HbA1c and FPG) were higher in the highest NHDLC/HDLC ratio tertile than the lowest tertile. Similar results were reported in a previous study [24]. As expected, the incidence of obesity notably increased across NHDLC/HDLC tertiles. The prevalence of NAFLD increased with increasing tertiles (NHDLC/HDLC ratio). The binary logistic regression analysis suggested that a higher NHDLC/HDLC ratio was positively correlated to NAFLD. In terms of sex distribution, the frequency of NAFLD by sex was significantly higher in boys than girls. The results of this study complies with Brunt et al. [25] and Welsh et al. [26]. This phenomenon may be attributed to the potential protective role of estrogen against hepatic steatosis [27, 28].

The findings here have certain clinical implications. The AUC results suggest that the NHDLC/HDLC ratio may be exercised as a more suitable indicator for NAFLD in childhood and adolescence than NHDLC and HDLC, especially in boys. Moreover, this study also assessed the diagnostic value of the NHDLC/HDLC ratio for NAFLD at different levels of ALT, and the results showed that the NHDLC/HDLC ratio was useful even in subjects with normal ALT levels. In addition, the NHDLC/HDLC ratio exhibits simplicity and feasibility for determination. Therefore, the NHDLC/HDLC ratio is feasible for screening and identifying NAFLD in children and adolescents.

### Study strengths and limitations

The conclusions of this large sample study are more convincing and meaningful. It was the first study to find that the NHDLC/HDLC ratio was positively associated with NAFLD in children and adolescents in China. The NHDLC/HDLC ratio exhibits simplicity and feasibility for determination, accordingly, the NHDLC/HDLC ratio may be helpful for identifying NAFLD in children and adolescents.

Some limitations are worth noting here. First, the NAFLD diagnosis was performed using ultrasound instead of liver biopsy to avoid the complications and invasiveness of liver biopsy. However, the NAFLD sensitivity of ultrasonography is reduced when the body fat percentage is < 30%, which may lead to misdiagnosis [29]. Second, since this was a single-center study, multicenter studies should be performed to confirm the conclusions. Third, this study was a cross-sectional study, therefore, the causal relationship between the NHDLC/HDLC ratio and NAFLD cannot be determined as a whole. This correlation needs further confirmation form prospective study. Fourth, since this study was performed only in Chinese children and adolescents, the association between the NHDLC/HDLC ratio and NAFLD needs to be confirmed in populations from other regions and ethnicities.

## Conclusions

The NHDLC/HDLC ratio is positively correlated with NAFLD in Chinese children and adolescents. It may be an effective indicator to help identify NAFLD in childhood and adolescence and prevent disease progression.

## Data Availability

All data used in this study are available from the corresponding author.

## Abbreviations

NHDLC/HDLC: Non-HDL-cholesterol to HDL-cholesterol
NAFLD: Non-alcoholic fatty liver disease
CLD: Chronic liver disease
NASH: Nonalcoholic steatohepatitis
MS: Metabolic syndrome
DM: Diabetes mellitus
CVD: Cardiovascular disease
IR: Insulin resistance
HDLC: High-density lipoprotein cholesterol
NHDLC: Non-high-density lipoprotein cholesterol
BMI: Body mass index
TC: Total cholesterol
FPG: Fasting plasma glucose
TG: Triglycerides
ALT: Alanine aminotransferase
ALP: Alkaline phosphatase
HbA1c: Glycated hemoglobin A1c
LDLC: Low-density lipoprotein cholesterol
ALB: Albumin
UA: Uric acid
AST: Aminotransferase
GGT: Gamma-glutamyl transferase
TB: Total bilirubin
Cr: Creatinine
HOMA-IR: Homeostasis Model Assessment of Insulin Resistance
OR: Odds ratio
IQR: Interquartile ranges
CI: Confidence intervals
ROC: Receiver operating characteristic
AUC: Area under the curve
FFAs: Free fatty acids
